# Structure and Magnetic Properties of SrFe_12−x_In_x_O_19_ Compounds for Magnetic Hyperthermia Applications

**DOI:** 10.3390/ma16010347

**Published:** 2022-12-30

**Authors:** Polina I. Nikolenko, Timur R. Nizamov, Igor G. Bordyuzhin, Maxim A. Abakumov, Yulia A. Baranova, Alexander D. Kovalev, Igor V. Shchetinin

**Affiliations:** Department of Physical Materials Science, University of Science and Technology MISIS, Leninsky Prospekt 4s1, 119049 Moscow, Russia

**Keywords:** magnetic hyperthermia, hexaferrite, SLP (specific power loss), ILP (intrinsic power loss), saturation magnetization, coercivity, doping, citrate synthesis

## Abstract

In this work, complex studies of the structure and magnetic properties of SrFe_12−x_In_x_O_19_ powders obtained by the mechanochemical and citrate methods were carried out. The solubility of In in strontium hexaferrite SrFe₁₂O₁₉ at 1200 °C was determined. The structure and properties of the powders were studied using X-ray diffraction analysis, Mössbauer spectroscopy and scanning electron microscopy. Measurements of magnetic properties in magnetic fields up to 1600 kA/m were also performed. Additionally, the hyperthermia effect was investigated. The possibility of controlling the coercivity of the samples in the range from 188.9 kA/m to 22.3 kA/m and saturation magnetization from 63.5 A·m^2^/kg to 44.2 A·m^2^/kg with an increase in the degree of In doping was also demonstrated. Investigation of the magnetic hyperthermia of the samples was carried out by temperature measurement with an IR camera when they were introduced into alternating magnetic fields of various frequencies (144, 261 and 508 kHz) and amplitudes (between 11.96 and 19.94 kA/m). According to the study result, there was detected the heating of the SrFe_12−x_In_x_O_19_ sample (x = 1.7). The highest values of magnetic hyperthermia of the sample were observed in a 19.94 kA/m magnetic field and a frequency of 261 kHz. At a concentration of 56.67 g/L, the sample was heated from 23 to 41 °C within 2 min. The parameters SLP (specific loss power) and ILP (intrinsic loss power) were calculated.

## 1. Introduction

Hard magnetic ferrites with a magnetoplumbite structure have been known since the middle of the 20th century [1]. From the moment of discovery to the present day, due to the unique combination of properties, hexaferrites have been widely used as materials for permanent magnets [2], microwave technology [3,4], information carriers [5,6], magneto-optics [7,8], absorbing materials [9], electronics and telecommunications [10], etc. Hexagonal ferrites can be obtained by a variety of methods such as ceramic synthesis [2], mechanochemical [11,12,13], co-precipitation [14,15,16], crystallization of oxide glass [17,18], hydrothermal [19,20,21], from salt melt [22], sol-gel [23,24], etc.

Other iron-based oxide materials, such as magnetite, maghemite and hematite, have been well studied in the literature for biomedicine applications as contrast agents for MRI [25,26,27], local hyperthermia [28,29], targeted drug delivery [30], in magnetic sensors and magnetic refrigerants [31]. Hexaferrites also belong to oxide materials and, in comparison with iron oxides, have a number of advantages [32,33,34,35]. At present time, the attention of scientists is attracted to research related to the biomedical applications of hexaferrites for diagnostics and therapy, and, in particular, for hyperthermia. There are studies that testify to the non-toxic nature of hexaferrites. Thus, it was demonstrated in [36] that the implantation of barium hexaferrite in tumors of tumor-bearing mice does not have a toxic effect on most organs.

Most of the research related to the magnetic hyperthermia is associated with stable colloidal suspensions of magnetic nanoparticles. Strontium hexaferrites SrFe_12_O_19_ are among the few hard magnetic materials based on nanoparticles of which it was possible to obtain stable colloidal solutions. The possibility of obtaining colloidal solutions of strontium hexaferrite was demonstrated in the studies [18,37,38]. This body of evidence indicates the potential of strontium hexaferrites as agents for magnetic hyperthermia in biomedical applications.

There are the following two main mechanisms for heating magnetic particles: hysteresis losses and relaxation. In multidomain particles, the main heating mechanism is hysteresis losses due to magnetization reversal. In single-domain particles, the main mechanism is relaxation, which can take place via the following two pathways: Neel relaxation is the rotation of magnetic moments inside the domain; Brown’s relaxation is the rotation of the body of the nanoparticle in the liquid.

The efficiency of magnetic particle heating Increases with higher frequency and amplitude of the applied magnetic field. However, there are restrictions on the maximum allowable safe amplitude-frequency product of the field. This is due to eddy currents, which, when using alternating magnetic fields, occur in all tissues, both affected and healthy. The estimated maximum values of the amplitude-frequency product, according to the Brezovich constraint, are given at the level of H_0f_ = 4.85 × 10^8^ [39].

In high-frequency fields with an admissibly low amplitude, hard magnetic materials with a high coercivity experience have almost no hysteresis losses and do not emit heat [40]. In this regard, the study of the doping effect on the formation of the structure and properties of strontium hexaferrites in order to adapt and use for hyperthermia is an urgent task.

## 2. Materials and Methods

In this work, the mechanochemical method was used to study the solubility limit of In in the SrFe_12_O_19_ compound and the compositions and the citrate method was used to synthesize samples to study the functional properties.

Strontium carbonate SrCO_3_ (99.9%) and Fe_2_O_3_ (99.9%), In_2_O_3_ (99.9%) oxides were used for the mechanochemical method, where they were mixed in certain quantities to obtain a hexaferrite phase, according to the following chemical reaction:

2 SrCO_3_ + (12–− x) Fe_2_O_3_ + x In_2_O_3_ → 2 SrFe_12−x_In_x_O_19_ + 2 CO_2_, where x = 0, 0.25, 0.5, 1, 3.

The amount of SrCO_3_ powder was between 1.43 and 1.22 g, the amount of Fe_2_O_3_ varied from 18.57 to 11.89 g and the mass of In_2_O_3_ was taken from 0 to 6.89 g. Total mass of the powder mixture per vial was 20 g. Then the powder mixtures were subjected to mechanochemical synthesis in an «Activator 2S» (Novosibirsk, Russia) planetary ball mill for 3 h at a rotation speed of 200/400 rpm (disk/vials). The ball-to-powder ratio was 1:10; steel balls and steel vials were used for milling. After mechanochemical synthesis the samples were annealed at 1200 °C for 1 h. The solubility limit was determined by changing the lattice spacings and the unit cell volume of the SrFe_12−x_In_x_O_19_ compound.

Aqueous solution of Sr(NO_3_)_2_, Fe(NO_3_)_3_ and In(NO_3_)_3_ was used SrFe_12−x_In_x_O_19_ for the citrate method synthesis. Then water was evaporated at 95 °C and the annealing was performed at 1200 °C (1 h in air).

The X-ray diffraction (XRD) phase analysis of SrFe_12−x_In_x_O_19_ compounds was carried out on a Rigaku Ultima IV (Tokyo, Japan) diffractometer with CoKα radiation and a graphite monochromator on a diffracted beam. XRD studies were carried out on powders that were placed into round cuvettes, and their surface was aligned with glass. The real degree of alloying of the samples obtained by the citrate method was determined from the change in the lattice spacings of the SrFe_12−x_In_x_O_19_ phase. The particle morphology of the samples was studied by scanning electron microscopy (SEM) in secondary electrons using a JEOL JSM-IT500 (Tokyo, Japan) electron microscope. The magnetic properties of the samples were measured on a VSM-250 (Changchun, China) vibrating sample magnetometer in fields up to 1600 kA/m. The powder samples were placed into copper cylinders, which were filled with paraffin. Then they were attached to a VSM rod vibrating during the measurements in an external magnetic field between the poles of electromagnet (in opened magnetic flux).

The study of magnetic hyperthermia of the samples was carried out in an aqueous medium with a small addition of sodium citrate (100 μL of 5% sodium citrate solution per 2 mL of water). The heating of the samples after applying the alternating magnetic field into the chamber was recorded by an infrared camera. Based on the study results, the *SLP* (specific loss power) and *ILP* (intrinsic loss power) parameters were calculated. The calculation was carried out in accordance with expressions (1) and (2) as follows:

(1) $$SLP=\frac{m_{sol}}{m_{part}}\cdot \frac{\Delta T}{\Delta t}\cdot C$$

where *SLP* is the specific power loss in W/g, *m_sol_* is the mass of the sol in kg, *m_part_* is the mass of particles in g, *C* is the heat capacity of the medium (for water 4183 J/(kg·K)), ∆*T* is the change of temperature in °C or K, ∆*t*—time in s.

(2) $$ILP=SLP\cdot \left(\frac{f}{H^{2}}\right)$$

where *ILP* is the intrinsic power loss in (nH·m^2^)/kg, *SLP* is the specific power loss in W/kg, *f* is the frequency of the magnetic field in kHz, *H* is the field strength in kA/m.

## 3. Results and Discussion

**Figure 1:**
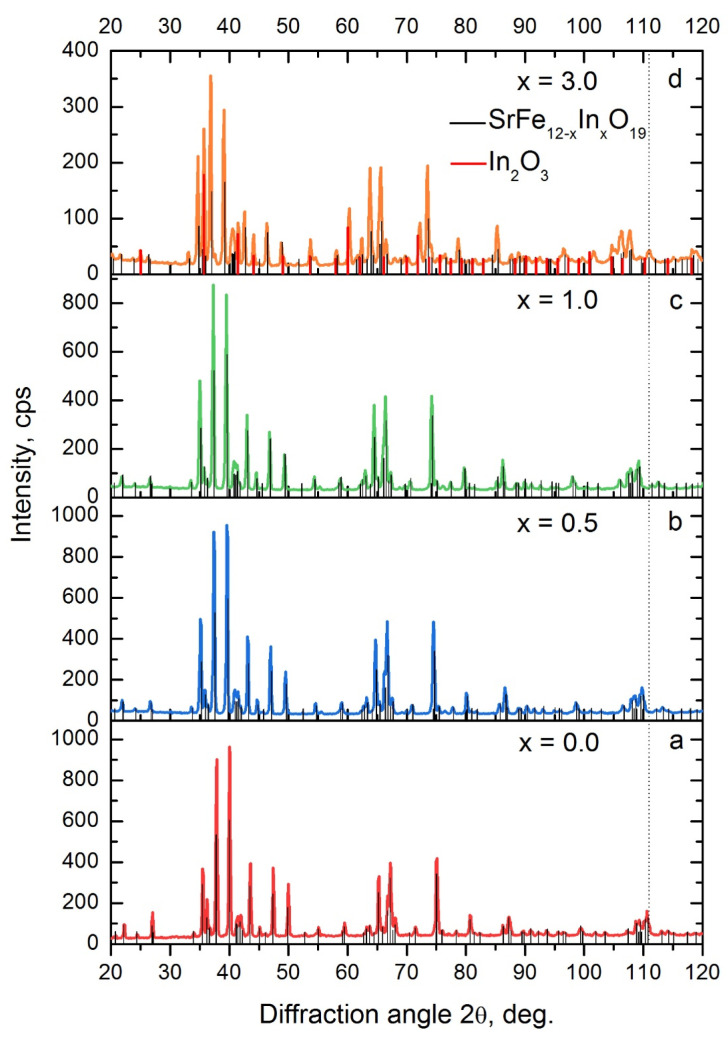
XRD patterns of samples SrFe_12−x_In_x_O_19_ after mechanochemical synthesis and annealing at 1200 °C, 1 h: x = 0 (**a**); x = 0.5 (**b**); x = 1.0 (**c**); x = 3.0 (**d**).

Samples of SrFe_12−x_In_x_O_19_, where x = 0, 0.25, 0.5, 1, 3 (at. In per formula unit), were obtained by mechanochemical synthesis and annealed at 1200 °C for 1 h. The XRD spectra and results of its analysis are presented in Figure 1 and Table 1.

**Figure 2:**
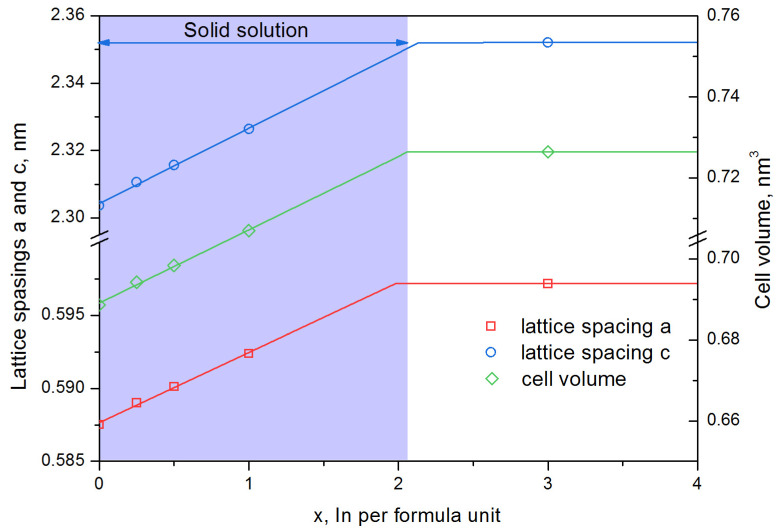
Dependence of lattice spacings and cell volume of the SrFe_12−x_In_x_O_19_ phase on the degree of alloying by In, after annealing at 1200 °C for 1 h.

According to Table 1, a graph of changes in hexaferrite lattice spacings was plotted as a function of the In doping degree (Figure 2). The phase lattice spacings change in the single-phase area, and upon transition to the two-phase/multiphase region, they become constant. Since in the two-phase/multiphase region a mixture of phases of the limiting composition is in equilibrium. Indium alloying leads to an increase in the lattice spacings and cell volume (Figure 2). The lattice spacings of the solid solution increase up to x ≈ 2.1 and reach a constant level above x ≈ 2.1. The cell volume of the phase behaves similarly with an increase in the atomic percentage of indium per formula unit. Since the In^3+^ ion radius (94 pm) is greater than the Fe^3+^ radius (69 pm) [41], the dissolution of In in the SrFe_12−x_In_x_O_19_ phase should lead to an increase in the lattice spacing and cell volume, which is observed in Figure 2. The point of intersection of the rising line and the constant value from the two-phase area corresponds to the maximum solubility of In in the SrFe_12−x_In_x_O_19_ phase at a temperature of 1200 °C. As shown in Figure 2 the maximum solubility of In in the SrFe_12−x_In_x_O_19_ phase at 1200 °C is x ≈ 2.1.

**Figure 3:**
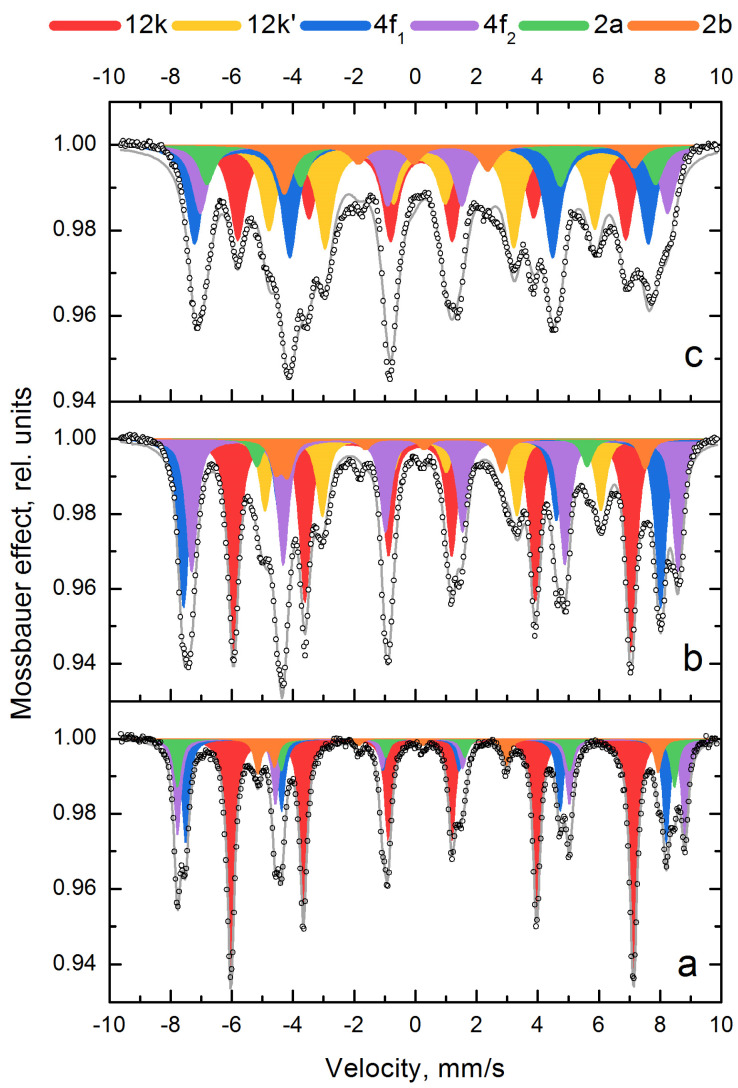
Mössbauer spectra of SrFe_12−x_In_x_O_19_ samples after mechanochemical synthesis and annealing at 1200 °C, 1 h with different degrees of Indium alloying: x = 0 (**a**); x = 0.5 (**b**); x = 1.0 (**c**). Circle – experimental data, grey line – calculated data.

According to Mössbauer spectroscopy (Figure 3, Table 2), the spectrum of non-alloyed strontium hexaferrite shows five sextets corresponding to five nonequivalent positions of iron in the structure. In the hexaferrite structure, iron ions occupy the following five non-equivalent sites: three of them are octahedral (12k, 2a and 4f_2_), one is tetrahedral (4f_1_) and one is trigonal bipyramidal (2b). Alloying of SrFe_12−x_In_x_O_19_ phase with indium leads to a change in the superfine parameters of the sextets (Table 2) and the appearance of the sixth sextet 12k’ (yellow), the share of which in the total spectrum increases with increasing degree of doping of the phase, and the share of the 12k (red) sextet in the total spectrum decreases, and their sum remains at the 50% level. The appearance of the 12k’ (yellow) sextet can be associated with a change in the immediate environment in the 12k (red) position due to the occupation of this position by indium. Moreover, when doping with indium, a decrease in the parameters of the hyperfine magnetic field of the sextets is observed, which may be associated with a decrease in the magnetocrystalline anisotropy constant [42].

Estimating the Mössbauer parameters in Table 2, it can be noted that there are the changes in the isomeric shift (I_S_) of the Fe^3+^ ions for all positions (sextets 1–6). The results of the analysis show high I_S_ values for the Fe^3+^ ions in the octahedral positions 12k, 4f_2_ and 2a (I_S_ = 0.33–0.46 mm/s) compared to the tetrahedral 4f_1_ (I_S_ = 0.15–0.26 mm/s). This can be explained by the greater covalence of bonds between iron ions due to the smaller volume of 4f_1_ polyhedra. In this case the quadrupole splitting Q_S_ of the Fe^3+^ ions in the 12k’ and 2b positions increases due to the larger deviation of the symmetry from the ideal state.

**Figure 4:**
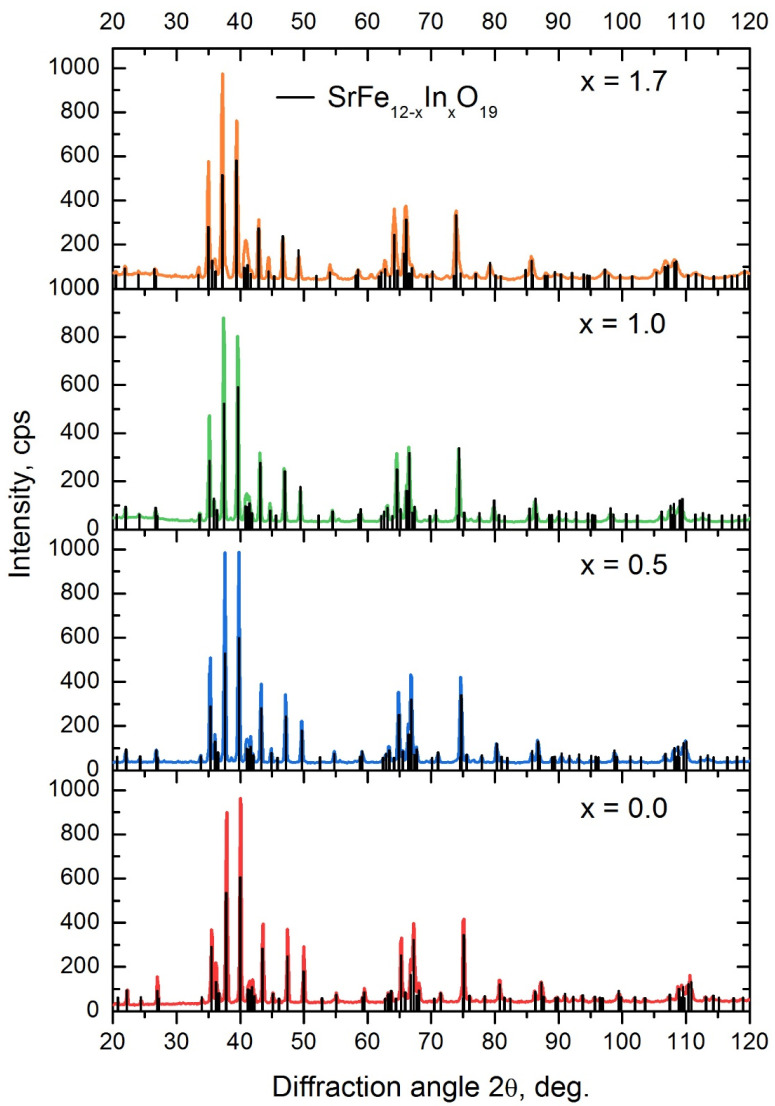
XRD patterns of SrFe_12−x_In_x_O_19_ samples obtained by the citrate method and annealed at 1200 °C for 1 h.

The obtained data are in good agreement with the results of the works [41,43]. Since chemical synthesis can lead to heterogeneity, and the activity of the components is different, the resulting linear dependence (Figure 2) was used to determine the real In content in the SrFe_12−x_In_x_O_19_ phase obtained by the citrate method. The citrate method was used to synthesize samples in a wide range of indium concentrations (x = 0–2), samples C1–C4 (Table 3). Samples C1–C3 with the degree of Indium alloying x ≤ 1 corresponded to the composition of the charge, and in sample C4 an underestimated content of indium was observed, which is associated with the formation of the In_2_O_3_ phase. The results of the phase analysis of SrFe_12−x_In_x_O_19_ samples obtained by the citrate method and annealed at 1200 °C for 1 h are presented in Table 3 and Figure 4. The presented data confirm that the synthesized samples contain the SrFe_12−x_In_x_O_19_ phase.

By the XRD analysis data, the samples obtained by the citrate method with x = 0–1.0 were in a single-phase state. Additionally, there were small XRD peaks observed in the sample with x = 1.7, coinciding with the position of the In_2_O_3_ peaks. The estimated amount of In_2_O_3_ was 2%.

**Figure 5:**
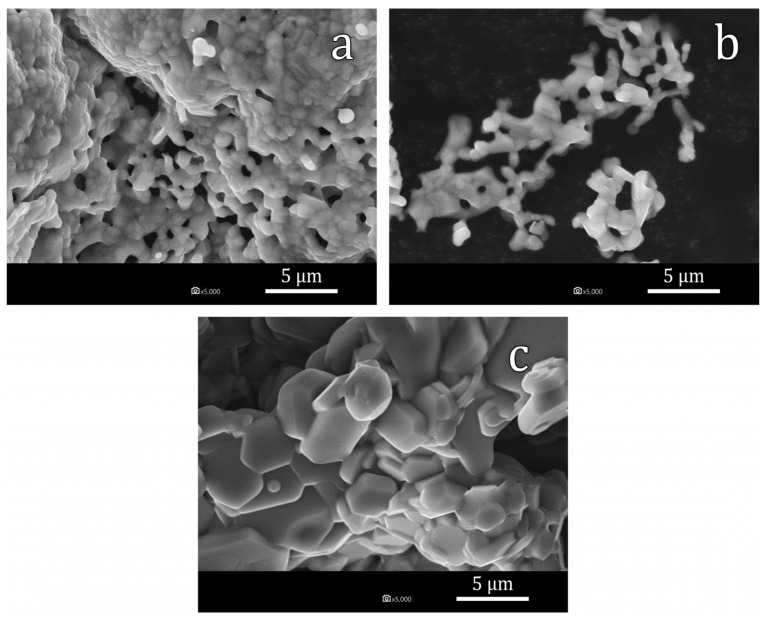
SEM images of SrFe_12−x_In_x_O_19_ particles, where x = 0.5 (**a**), x = 1.0 (**b**), x = 1.7 (**c**), obtained by the citrate method and annealed at 1200 °C for 1 h.

The obtained photographs of the particles of SrFe_12−x_In_x_O_19_ samples C2 (x = 0.5), C3 (x = 1.0) and C4 (x = 1.7) are shown in Figure 5. According to the SEM data, it can be seen that the particles have a size of 0.5–5 µm. Figure 5 shows that at low doping levels C2 and C3 (x = 0.5 and x = 1.0), the average particle size does not generally exceed 2.5 µm, while in the photo of the C4 (x = 1.7) sample, the particle size is at the level of 5 µm, which is due to influence of In and is consistent with the results of the work [43]. Moreover, it can be suggested by the XRD spectra analysis that with an increase in the degree of doping by indium, an increase in lattice microstrain occurs. This fact can be associated with the appearance of defects in the crystal structure and also with the effective increase in the diffusion coefficient.

**Figure 6:**
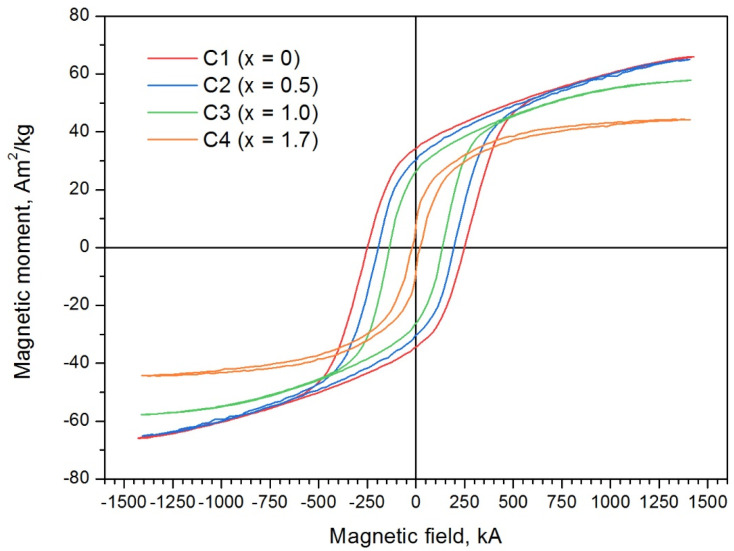
Hysteresis loops and magnetic properties of SrFe_12−x_In_x_O_19_ samples obtained by the citrate method and annealed at 1200 °C for 1 h.

The magnetic hysteresis loops and the magnetic parameters of the SrFe_12−x_In_x_O_19_ samples obtained by the citrate method are shown in Figure 6 and Table 4. Hysteresis loops show that alloying with In leads to a fast decrease in the coercivity to 22.3 kA/m and magnetization to 42.95 A·m^2^/kg. This behavior of the magnetic properties is due to the fact that In generally occupies the 12k position, which, along with the 2b position, is responsible for the magnetocrystalline anisotropy constant [43,44], which leads to a decrease in the magnetocrystalline anisotropy constant and of the coercivity, respectively. Moreover, the coercivity decrease can be due to the grain growth of the SrFe_12−x_In_x_O_19_ phase, which is observed in a highly alloyed sample C4 (x = 1.7). In the structure of strontium hexaferrite, the magnetic moments of the Fe^3+^ ions in positions 2a, 2b and 12k are directed ferromagnetically, and in positions 4f_1_ and 4f_2_—antiferromagnetically [2]. When the nonmagnetic In^3+^ ion substitutes the Fe^3+^ in the 12k position, the total ferromagnetic moment in the cell decreases as well as the magnetization of the whole sample [43]. It should be noted that In doping makes it possible to control the magnetic properties of the samples over a wide range. For use materials in hyperthermia, the coercivity must be less than the amplitude of the high-frequency field to emit the remagnetization losses in the form of heat.

**Figure 7:**
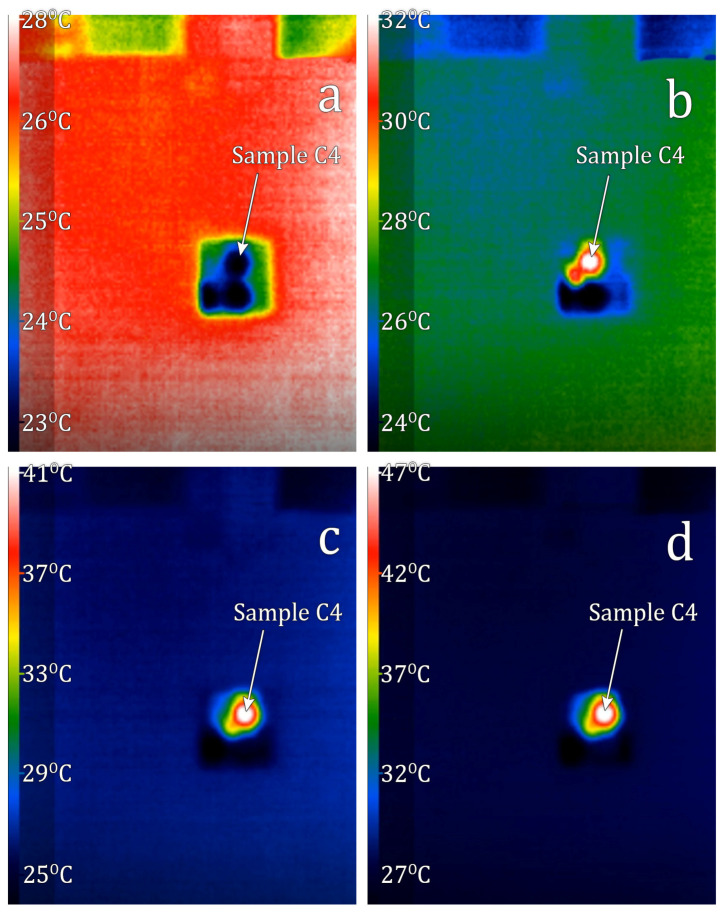
Temperature of sample C4 as time function: (**a**)—0 min.; (**b**)—1 min.; (**c**)—2 min.; (**d**)—3 min.

SrFe_12−x_In_x_O_19_ samples C2 (x = 0.5) and C3 (1.0), did not experience visible heating when introduced into a low-amplitude alternating magnetic field. For the sample with the lowest coercivity, SrFe_12−x_In_x_O_19_ C4 (x = 1.7), when introduced into an alternating magnetic field evident heating was observed. Sample temperature changes depending on the experiment time are shown in Figure 7. Video of these sample temperature changes vs time can be accessible in Supplementary materials to this paper. The results of measured and calculated SLP and ILP parameters for this sample are shown in Table 5. Indium alloying leads to a decrease in the coercivity to the level of high-frequency field amplitude used for medical purposes. This causes a reversal magnetization of the particles by these fields and heat generation. The obtained values correspond to the level of properties of the materials researched at the present time [45,46].

## 4. Conclusions

1. The solubility limit of In in hexaferrite SrFe_12_O_19_ at 1200 °C was determined—it reaches to x = 2.1 In atoms per hexaferrite formula unit. According to Mössbauer spectroscopy data, In predominantly occupies the 12k positions;
2. Using the citrate method and subsequent annealing at 1200 °C, the SrFe_12−x_In_x_O_19_ powders with x = 0.5, x = 1.0 and x = 1.7 were obtained. All synthesized samples contained at least 97% of the SrFe_12−x_In_x_O_19_ phase. Sample particles had sizes in the range of 0.5–5 µm and were collected in agglomerates. As the degree of doping increases, an increase in the average particle size is observed;
3. As the degree of substitution of iron by indium increases from x = 0.5 to x = 1.7, the coercivity of the samples strongly decreases from 188.9 kA/m to 22.3 kA/m. The specific magnetization decreases from 63.5 A·m^2^/kg to 44.2 A·m^2^/kg;
4. SrFe_12−x_In_x_O_19_ sample with x = 1.7 experienced evident heating when introduced into an alternating magnetic field. The best results of magnetic hyperthermia of the sample were observed at a field strength of 19.94 kA/m and a frequency of 261 kHz (at a high sample concentration (56.67 g/L), heating from 23 to 41 °C was achieved in 2 min).

## Figures and Tables

**Table 1:** The XRD results of SrFe_12−x_In_x_O_19_ samples obtained by mechanochemical synthesis followed by annealing at 1200 °C for 1 h.

x, at. in per Formula Unit	Phase Composition, % (±3)	Phase SrFe_12−x_In_x_O_19_ Parameters			
		a, nm (±0.0001)	c, nm (±0.0003)	Cell Volume, nm^3^ (±0.003)	c/a Ratio (±0.001)
0	SrFe_12_O_19_—100%	0.5875	2.3036	0.6886	3.921
0.25	SrFe_12−x_O_19_—100%	0.589	2.3106	0.6942	3.923
0.5	SrFe_12−x_O_19_—100%	0.5901	2.3157	0.6984	3.924
1.0	SrFe_12−x_O_19_—100%	0.5924	2.3263	0.7070	3.927
3	SrFe_12−x_O_19_—83% In_2_O_3_—17%	0.5972	2.352	0.7264	3.939

**Table 2:** Mössbauer spectra parameters of samples SrFe_12−x_In_x_O_19_ after mechanochemical synthesis and annealing at 1200 °C, 1 h.

Hyperfine Parameters of Mössbauer Spectra	Sample	Fe Site					
		12k	12k’	4f_1_	4f_2_	2a	2b
Hyperfine magnetic field H_hf_, kOe	x = 0	409	-	488	515	505	406
	x = 0.5	403	341	484	494	488	378
	x = 1.0	394	330	461	474	455	355
Isomer shift (I_S_), mm/s	x = 0	0.35		0.26	0.37	0.34	0.29
	x = 0.5	0.35	0.31	0.15	0.46	0.42	0.35
	x = 1.0	0.37	0.33	0.19	0.46	0.51	0.24
Quadrupole splitting (Q_S_), mm/s	x = 0	0.40	-	0.17	0.29	0.02	2.2
	x = 0.5	0.39	0.37	0.11	0.39	0.08	2.2
	x = 1.0	0.34	0.40	0.00	0.30	0.02	2.4
Relative intensity, %	x = 0	48	-	19	18	9	6
	x = 0.5	35	14	18	20	7	6
	x = 1.0	26	24	21	15	8	6

**Table 3:** Phase analysis of SrFe_12−x_In_x_O_19_ samples obtained by the citrate method and annealed at 1200 °C for 1 h.

Sample	x, at. in per Formula Unit (Estimated)	Phase Composition, %	Phase SrFe_12−x_In_x_O_19_ Parameters			
			a, nm (±0.0001)	c, nm (±0.0003)	Cell Volume, nm^3^ (±0.03)	c/a Ratio (±0.001)
C1	0	SrFe_12_O_19_—100%	0.5876	2.3034	0.6888	3.920
C2	0.5	SrFe_12_O_19_—100%	0.5903	2.3144	0.6984	3.921
C3	1.0	SrFe_12_O_19_—99.5% Fe_2_O_3_—0.5%	0.5926	2.3264	0.7075	3.926
C4	1.7	SrFe_12_O_19_—98% In_2_O_3_—2%	0.5955	2.343	0.7196	3.935

**Table 4:** Magnetic properties of SrFe_12−x_In_x_O_19_ samples obtained by the citrate method and annealed at 1200 °C for 1 h.

Sample	x, at. in per Formula Unit (Estimated Quantity)	Coercitivity Force H_c_, kA/m	Remanence Magnetization σ_r_, A·m^2^/kg	Saturation Magnetization σ_s_, A·m^2^/kg
C1	0	254.1	65.3	33.5
C2	0.5	188.9	63.5	28.6
C3	1.0	135.6	57.7	26.2
C4	1.7	22.3	44.2	7.7

**Table 5:** Magnetic hyperthermia of the SrFe_12−x_In_x_O_19_ sample C4 (x = 1.7), obtained by the citrate method and annealed at 1200 °C for 1 h.

Concentration, g/L	Time, s	f, kHz	H, kA/m	T_start_, °C	T_finish_, °C	SLP, W/g	ILP, (nH·m^2^)/kg
10.00	120	261	19.94	20	22	6.97	0.067
56.67	120	261	19.94	23	41	11.07	0.107
56.67	120	508	11.96	22	31	5.54	0.076
56.67	120	144	19.94	21	27	3.69	0.064

## Data Availability

Not applicable.

## Supplementary Materials

The following supporting information can be downloaded at: https://www.mdpi.com/article/10.3390/ma16010347/s1. Video S1: Sample temperature changes depending on the experiment time.
